# Breast cancer risk assessment for screening: a hybrid artificial intelligence approach

**DOI:** 10.1007/s00330-025-11980-9

**Published:** 2025-09-11

**Authors:** Raquel Tendero, Andrés Larroza, Francisco Javier Pérez-Benito, Juan Carlos Perez-Cortes, Marta Román, Rafael Llobet

**Affiliations:** 1 Instituto Tecnológico de la Informática, Universitat Politècnica de València, Camino de Vera s/n, 46022 València, Spain; 2 Universitat Politècnica de València, Camino de Vera s/n, 46022 València, Spain; 3 Department of Epidemiology and Evaluation, IMIM (Hospital del Mar Research Institute), Passeig Marítim 25-29, 08003 Barcelona, Spain

**Keywords:** Breast neoplasm, Mammography, Artificial intelligence, Risk assessment, Screening

## Abstract

**Objectives:**

This study evaluates whether integrating clinical data with mammographic features using artificial intelligence (AI) improves 2-year breast cancer risk prediction compared to using either data type alone.

**Materials and methods:**

This retrospective nested case-control study included 2193 women (mean age, 59 ± 5 years) screened at Hospital del Mar, Spain (2013–2020), with 418 cases (mammograms taken 2 years before diagnosis) and 1775 controls (cancer-free for ≥ 2 years). Three models were evaluated: (1) ERTpd + im, based on Extremely Randomized Trees (ERT), split into sub-models for personal data (ERTpd) and image features (ERTim); (2) an image-only model (CNN); and (3) a hybrid model (ERTpd + im + CNN). Five-fold cross-validation, area under the receiver operating characteristic curve (AUC), bootstrapping for confidence intervals, and DeLong tests for paired data assessed performance. Robustness was evaluated across breast density quartiles and detection type (screen-detected vs. interval cancers).

**Results:**

The hybrid model achieved an AUC of 0.75 (95% CI: 0.71–0.76), significantly outperforming the CNN model (AUC, 0.74; 95% CI: 0.70–0.75; *p* < 0.05) and slightly surpassing ERT*pd + im* (AUC, 0.74; 95% CI: 0.70–0.76). Sub-models ERT*pd* and ERT*im* had AUCs of 0.59 and 0.73, respectively. The hybrid model performed consistently across breast density quartiles (*p* > 0.05) and better for screen-detected (AUC, 0.79) than interval cancers (AUC, 0.59; *p* < 0.001).

**Conclusions:**

This study shows that integrating clinical and mammographic data with AI improves 2-year breast cancer risk prediction, outperforming single-source models. The hybrid model demonstrated higher accuracy and robustness across breast density quartiles, with better performance for screen-detected cancers.

**Key Points:**

***Question*** *Current breast cancer risk models have limitations in accuracy. Can integrating clinical and mammographic data using artificial intelligence (AI) improve short-term risk prediction?*

***Findings**** A hybrid model combining clinical and imaging data achieved the highest accuracy in predicting 2-year breast cancer risk, outperforming models using either data type alone*.

***Clinical relevance**** Integrating clinical and mammographic data with AI improves breast cancer risk prediction. This approach enables personalized screening strategies and supports early detection. It helps identify high-risk women and optimizes the use of additional assessments within screening programs*.

**Graphical Abstract:**

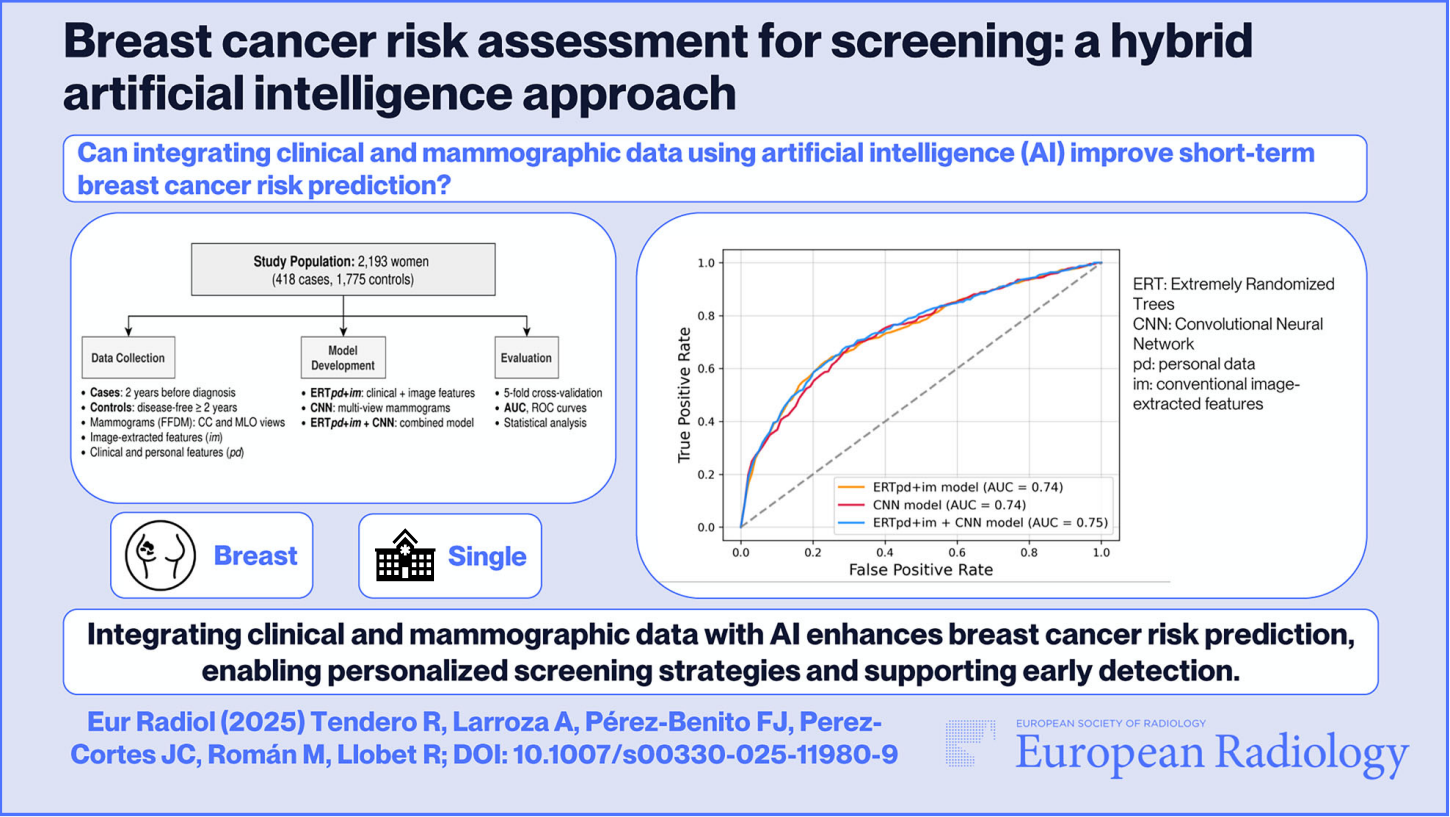

## Introduction

Mammographic screening is a standardized procedure for early breast cancer detection, which has been shown to reduce mortality from this disease [1, 2]. The interpretation of mammographic images is performed by trained radiologists and involves processing substantial volumes of data [3]. The continuous development of effective detection [4] and prediction techniques [5] is playing a crucial role in improving radiological effectiveness, with Artificial Intelligence (AI) establishing a new standard by significantly reducing the workload of radiologists [6]. Various AI tools have been developed to assist with image interpretation [7, 8], demonstrating an increase in diagnostic performance [9, 10]. However, the use of AI for risk prediction of women undergoing mammographic screening is an innovative approach [11], with limited application in clinical practice [12].

Several studies have shown that breast density is a well-established risk factor [13, 14], associating dense breast tissue with an increased probability of cancer development [15]. Other factors such as family history of breast cancer, previous benign breast disease, age, and gynecologic information have proven to be strong clinical predictors for breast cancer at 5 or 10 years [16, 17]. Despite this, there is a growing interest in leveraging additional information extracted from mammograms [18] to enhance risk assessment and combine it with traditional risk factors to accomplish more accurate and personalized results [19].

Long-term risk models (5–10 years) are valuable for developing population-based approaches as well as designing screening strategies. However, AI-based models have shown potential for short-term prediction, allowing for targeted assessments of women at high risk. This aligns with the standard screening interval in Europe, which typically ranges from two to three years [2]. Unlike long-term models, which primarily inform general screening policies, short-term models are particularly useful in identifying high-risk individuals who may benefit from earlier intervention through more frequent monitoring or additional diagnostic procedures.

This study aims to integrate clinical data with structural information obtained from mammograms to build risk prediction models based on machine learning techniques. We compared three machine learning-based predictive models to estimate the 2-year risk of developing breast cancer in women targeted for breast cancer screening. The first model (1) relies on personal data, risk factors, and conventional features extracted from mammograms. The second model (2) is based on Convolutional Neural Networks (CNN) that analyze mammograms exclusively. The final hybrid model (3) integrates all these data sources to assess whether combining clinical and mammographic data enhances risk prediction. This architecture allows us to compare traditional features with automatically learned patterns from full images, and to evaluate their combined contribution to short-term risk prediction.

## Materials and methods

This study was supported by grants from Instituto de Salud Carlos III FEDER (PI17/00047). The funders had no role in the study’s design, conduct, or reporting. The Ethics Committee at Hospital del Mar Medical Research Institute (IMIM) (2017/7442/I, approved on 14 December 2017) granted approval for the study. The study was conducted according to the guidelines of the Declaration of Helsinki. Informed consent from study participants was not required since we used anonymized retrospective data.

### Study population

The study comprised women aged 50–69 who underwent mammographic screening between 2013 and 2020 as part of the publicly funded breast cancer screening program at Hospital del Mar, Spain. The program follows the European Guidelines [2] and screens over 16,000 women annually, with a participation rate of 67% and a 91.2% [20, 21] re-attendance rate. In Spain, the national breast cancer screening program follows a biennial interval, in which women with negative assessments are invited for screening every two years. Individuals with a prior history of breast cancer were not included. This was a single-center study.

### Study design

The aim of this study was to evaluate AI-based models for predicting 2-year breast cancer risk using mammographic images and clinical data from a population-based screening program. Screening mammography was interpreted by double reading, with arbitration by a third radiologist in case of disagreement. This retrospective nested case–control study included women screened using full-field digital mammography (FFDM).

Cases were defined as screening mammograms assessed as negative at the time of acquisition, corresponding to patients who were subsequently diagnosed with breast cancer within two years. Diagnosis was confirmed by an anatomopathological report, using the SNOMED classification, to determine the presence of malignancy.

Screen-detected cancers were defined as breast malignancies diagnosed as a consequence of a positive assessment by screening mammography. Interval cancers were those diagnosed in the time interval between a negative screening test and before the next screening appointment, or in the two years after the last screening participation for the upper age limit of 69 and 70 years of age. Interval breast cancer cases were included through record linkage to the screening program’s data.

Controls were randomly selected from disease-free women observed for at least two years from image acquisition. They were frequency-matched at a 5:1 ratio by exact age (from 50 to 69 years) and imaging device to ensure comparability between cases and controls.

### Mammographic image and clinical data collection

**Fig. 1:**
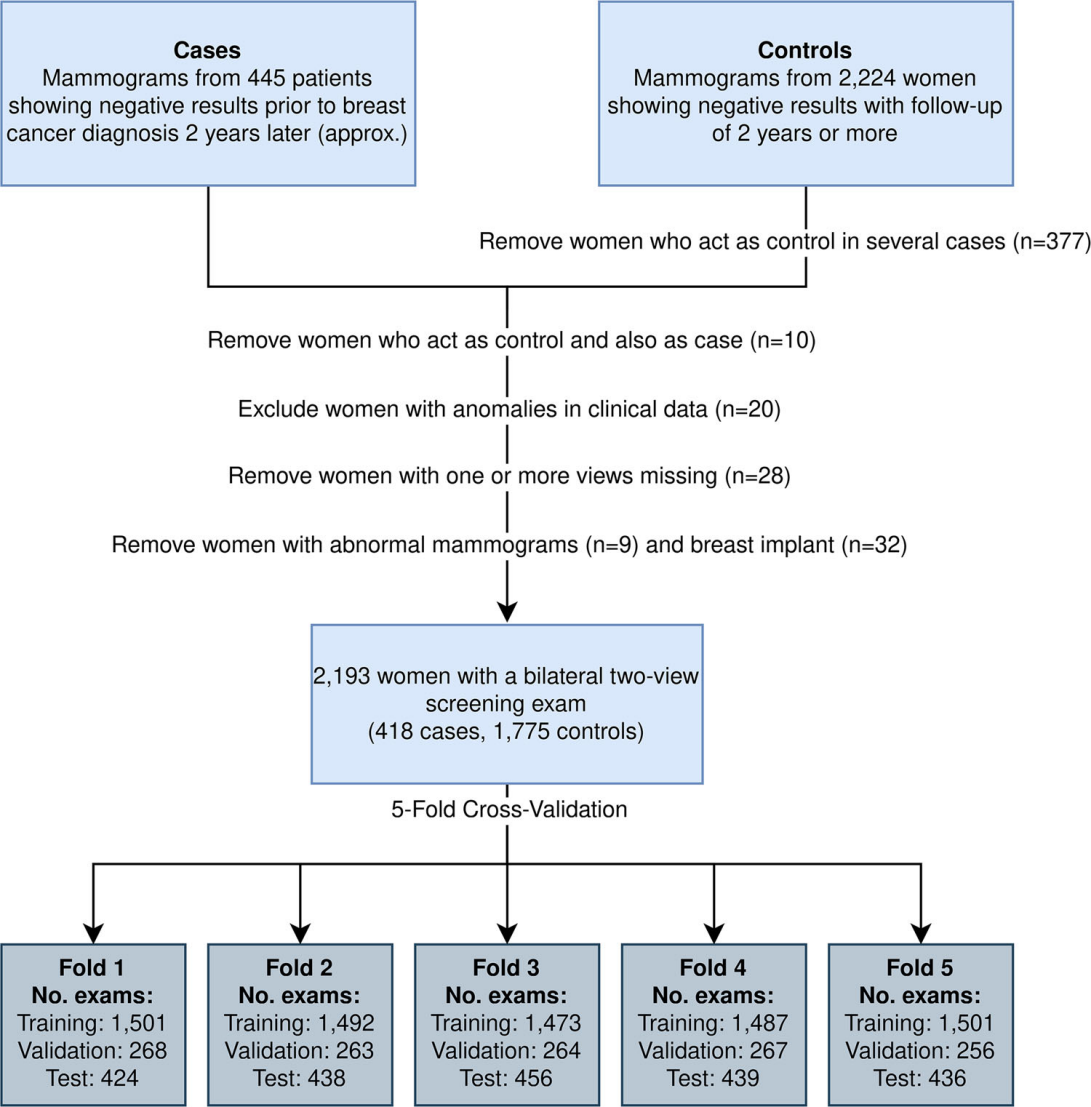
Data selection flowchart. It details the criteria for women’s inclusion/exclusion from the 2669 original exams obtained between 2013 and 2020. It also illustrates the 5-fold cross-validation approach used in the study

FFDM images for presentation (mediolateral oblique and craniocaudal views) of both breasts were obtained from the PACS system. Clinical risk factors and breast cancer status were collected from the breast cancer screening program databases. Mammograms and clinical data were anonymized to preserve data protection. The images were acquired using devices from General Electric and FUJI. We included all women screened between 2013 and 2020 with available clinical information and whose mammograms in DICOM format were also available. We excluded women acting as controls in multiple cases, those who initially acted as controls and later as cases in subsequent exams, and those with breast implants (Fig. 1).

We employed 5-fold stratified cross-validation to ensure robust model evaluation, with case–control pairings preserved within each subset. In each iteration, one-fold (20%) was used as an independent test set, while the remaining four folds (80%) were split into training (85%) and validation (15%) subsets. This procedure was repeated five times, ensuring that each fold served once as the test set. Performance metrics were averaged across the five test folds to obtain a comprehensive model evaluation.

### Data preprocessing

We applied pixel normalization [22] and SAM-breast [23] to eliminate non-breast areas. Images were resized to 256 × 256 pixels. Since the original mammograms varied in size and were not square, we first applied padding with background values to produce square images prior to resizing. This approach preserved anatomical proportions and avoided distortion. These preprocessing steps were applied before feature extraction, ensuring that both conventional image features and CNN-based models worked with the same processed images. For a more generalized model, we used data augmentation [24] using the Albumentations library (version 1.4.0; albumentations.ai) in Python (version 3.8.0; python.org). Optical and grid distortions, random size clippings, and Gaussian noise were applied to the training images.

Regarding clinical data, we filled in missing values using median imputation or *k*-nearest neighbors, depending on the variable, and handled atypical entries. For mammographic images, we extracted conventional features, including geometric variables and intensity-based measurements, from both the entire breast and the dense tissue (DT) area using the ndimage package (version 1.10.1; scipy.org) and the measure module (version 0.21.0; scikit-image.org). DT was obtained using the CM-YNet model [25]. After combining clinical and image features, we used SEQENS [26] to identify the most relevant variables, resulting in a final set comprising personal (*pd*) and image-extracted (*im*) data.

### Risk assessment models

We developed three AI-based models using two distinct architectures: Extremely Randomized Trees (ERT) [27] and Convolutional Neural Networks (CNN) [28]. The models evaluated were: **ERT*pd + im*** (along with ERT-based sub-models **ERTpd** and **ERTim),** **CNN**, and **ERT*pd + im ***+ **CNN**. A detailed description is available in the Electronic Supplementary Material.

**ERT*pd + im*** **model**: It uses the ERT classifier (version 0.24.2; scikit-learn.org) to predict the 2-year risk using personal data and image-extracted features. We also trained two sub-models for performance comparison: one on personal data (**ERT*pd****)*, and another on image data (**ERT*im****)*. Both sub-models use the same architecture and hyperparameters as the main model.

**CNN model**: It processes 256 × 256-pixel mammograms and predicts the risk using a multi-view CNN (version 2.6.2; tensorflow.org).

**ERT*pd + im ***+ **CNN model**: It is a hybrid model consisting of an ERT classifier that combines the personal and image data from the first model with features extracted from the penultimate layer of the second model, which summarizes crucial image characteristics for enhancing prediction.

We evaluated the models using the area under the curve (AUC) metric together with receiver operating characteristic (ROC) curves. To analyze performance across different densities, we categorized breasts into quartiles based on the average DT from the four projections. The quartile thresholds were derived empirically from the distribution of DT percentages in the study dataset. Additionally, we evaluated the models for different types of cancer according to detection method, calculating AUCs separately for screen-detected and interval cancers by comparing each subgroup against the control group. Furthermore, to assess the relative importance of each input feature in the hybrid model’s predictions, we generated feature importance plots using mean decrease impurity (MDI), and included Grad-CAM visualizations [29] showing examples of correctly classified cases and controls to illustrate the mammographic regions the CNN model focused on during prediction.

### Statistical analysis

To compare the models’ performance, we applied the DeLong test [30] to the ROC curves using the pROC package [31] (version 1.18.4) in R (version 4.3.1, R Core Team, https://www.R-project.org). We calculated the 95% confidence intervals using bootstrapping with 1000 iterations. Chi-squared and t-tests were used to compare variables across the case and control groups. *p*-values were considered statistically significant at *p* < 0.05.

## Results

### Patient characteristics

Initially, the case group comprised 445 women and the control group 2224. We excluded 377 women who acted as controls for more than one case, and ten controls who later developed the disease. Additionally, we excluded 20 women with significant missing or atypical data, nine with image abnormalities, and 32 with breast implants due to potential interference with image interpretation [32]. Finally, 28 women were excluded due to missing one or more views (Fig. 1).

Finally, the study population comprised a total of 2193 women (mean age ± standard deviation: 59 years ± 5), including 418 cases and 1775 controls. Information on the women’s clinical details and risk factors is summarized in Table 1. Previous benign breast disease was more common among cases (31.8% vs. 21%, *p* < 0.001), further emphasizing its relevance as a risk factor. Also, cases had a higher prevalence of first-degree relatives with breast cancer compared to controls (13.4% vs. 10.1%), even though this difference was not significant (*p* = 0.056). Furthermore, cases also had a significantly higher prevalence of second-degree relatives with a history of breast cancer (23.5% vs. 19%, *p* < 0.05). We found no evidence of a difference between cases and controls regarding age at menopause, hormone replacement therapy use, smoking status, or educational level (*p* > 0.05).

**Table 1:** Patient characteristics

Characteristic	Cases (*n *= 418)	Controls (*n* = 1775)
Age (years)^a^	59 ± 5	59 ± 5
Age at menopause (years)^a^	42 ± 18	43 ± 17
No. of first-degree relatives with breast cancer		
0	362 (86.6)	1595 (89.9)
1	52 (12.4)	171 (9.6)
≥ 2	4 (1.0)	9 (0.5)
Second-degree relatives’ history of breast cancer		
No	320 (76.5)	1438 (81.0)
Yes	98 (23.5)	337 (19.0)
Hormone replacement therapy ever used		
No	402 (96.2)	1701 (95.8)
Yes	16 (3.8)	74 (4.2)
Previous Benign Breast Disease		
No	285 (68.2)	1402 (79.0)
Yes	133 (31.8)	373 (21.0)
Smoking		
Never	220 (52.6)	998 (56.2)
Ever (ex-smoker)^b^	126 (0.74 ± 0.42) (30.2)	491 (0.72 ± 0.46) (27.7)
Current^b^	72 (0.695 ± 0.37) (17.2)	286 (0.61 ± 0.38) (16.1)
Education		
Low	143 (34.2)	634 (35.7)
Medium	163 (39.0)	739 (41.6)
High	112 (26.8)	402 (22.7)

Unless otherwise indicated, data are the number of women, with percentages in parentheses. Age is not included in the experiments since it is already controlled by case/control matching

^a^ Data are means ± standard deviations

^b^ Data are the number of women. The first parentheses indicate smoking packs per year (1 pack = 20 cigarettes per day), means ± standard deviations. The second parentheses indicates the percentage

Image acquisition parameters are summarized in Supplementary Table 1. Despite minor variations, the overall consistency in acquisition parameters supports the reliability and comparability of the imaging data between cases and controls.

### Data collection

After data preparation, the resulting dataset included three primary types of data. Personal data (pd), which encompasses demographic and clinical details of the participants, is summarized in Table 1. Image-extracted data (im), listing the conventional features extracted from the mammograms, is presented in Supplementary Table 2. For further details, Supplementary Tables 3–6 provide comprehensive descriptions of the variables for each of the four views. Finally, the third data type consists of preprocessed mammograms, which underwent specific transformations detailed in the Data Preprocessing section of Materials and Methods. Depending on the model, this input consists of either the full preprocessed mammogram (used in CNN) or a set of 64 extracted features (used in the hybrid model).

### Model performance evaluation

The ERT*pd + im* and CNN models showed equal AUCs of 0.74 (95% CI: 0.70, 0.76) and (95% CI: 0.70, 0.75), respectively (*p* = 0.72). The hybrid model achieved a higher AUC of 0.75 (95% CI: 0.71, 0.76), demonstrating superior performance compared to the CNN model (*p* = 0.02). Although this improvement is statistically significant, the magnitude of the difference is small. Additionally, we found no evidence of a significant difference relative to the ERT*pd + im* model (*p* = 0.19).

**Fig. 2:**
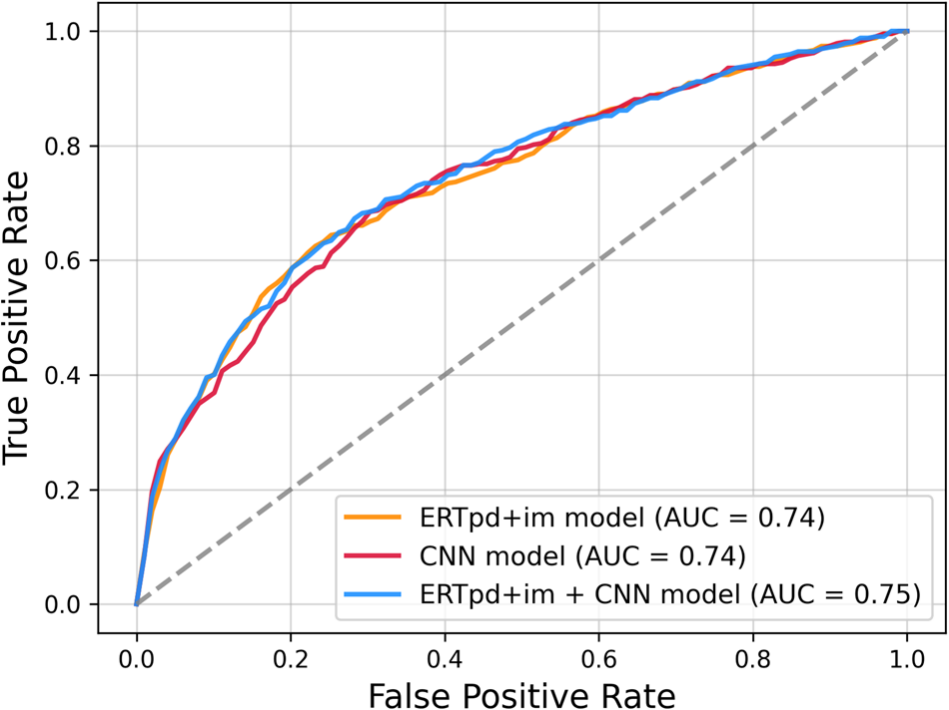
Receiver operating characteristic (ROC) curve of the ERTpd + im, CNN, and hybrid (ERTpd + im + CNN) models’ performance on the test set (pd = personal data, im = features extracted from images, ERT = Extremely Randomized Trees, CNN = Convolutional Neural Networks). Each model’s effectiveness is depicted by the mean AUC value, derived from the five test sets generated during the 5-fold cross-validation process

The performance evaluation is presented in Table 2, and the ROC curves are illustrated in Fig. 2. Despite some overlap in confidence intervals, the hybrid model demonstrated a slight but consistent performance improvement over the others.

**Table 2:** Performance evaluation of risk estimation models

Model	Mean AUC	CI (95%)
ERT*pd*	0.59 ± 0.04	[0.55, 0.61]
ERT*im*	0.73 ± 0.04	[0.69, 0.75]
ERT*pd* + *im*	0.74 ± 0.04	[0.70, 0.76]
CNN	0.74 ± 0.03	[0.70, 0.75]
ERT*pd*+*im* + CNN	**0.75** ± **0.03**	[0.71, 0.76]

Data are mean AUC ± standard deviations obtained during 5-fold cross-validation

*CI* confidence intervals calculated with bootstrapping over 1000 iterations, *ERT* Extremely Randomized Trees, *CNN* Convolutional Neural Network, *pd* personal data, *im* image-extracted features

Bold values indicate the maximum mean AUC across the compared models

Regarding the sub-models, ERT*pd* and ERT*im*, the performance of the model using only personal data had an AUC of 0.59, which was lower than that of the imaging model, which achieved an AUC of 0.73 (*p* < 0.05). However, there were no significant differences between ERT*im* and ERT*pd + im*, indicating that the addition of personal data to the imaging model yielded only a marginal improvement in AUC (from 0.73 to 0.74), suggesting limited complementary information.

### Subgroups according to dense tissue percentage

**Fig. 3:**
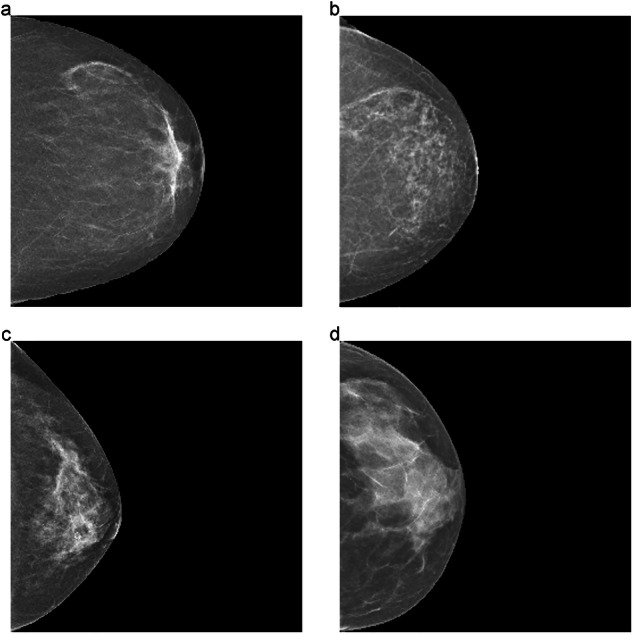
We provide visual examples of breast density percentages within quartiles (**a**) 5.3% (Q1), (**b**) 8.6% (Q2), (**c**) 15.7% (Q3), (**d**) 27.3% (Q4)

In order to analyze the results by DT percentage, the dataset was divided into quartiles as follows: Q1 (DT ≤ 7.25%), Q2 (7.25% < DT ≤ 12.11%), Q3 (12.11% < DT ≤ 17.96%), and Q4 (DT > 17.96%). Examples of each group are shown in Fig. 3.

The results presented in Table 3 show that all models evaluated had higher average AUC values in Q2. In Q4, consisting of the densest breasts, a slight decrease in the performance of all models was observed compared to other quartiles. However, we found no statistical evidence of differences between quartiles for any of the models (all *p* > 0.05). Regarding model comparison, the hybrid model outperformed the other models in the first three density-based quartiles. Additionally, the interval cancer rate increased with breast density, consistent with previous findings [33].

**Table 3:** Model performance by average breast dense tissue percentage

Dense tissue quartile	ERT*pd + im*	CNN	ERT*pd + im* + CNN	Interval cancer (%)
Q1 (DT ≤ 7.25%)	0.75 [0.65, 0.78]	0.75 [0.68, 0.80]	**0.76** [0.69, 0.80]	15.5
Q2 (7.25% < DT ≤ 12.11%)	**0.77** [0.70, 0.81]	0.76 [0.70, 0.81]	**0.77** [0.70, 0.82]	15.8
Q3 (12.11% < DT ≤ 17.96%)	0.73 [0.64, 0.77]	0.73 [0.64, 0.76]	**0.76** [0.69, 0.80]	20.4
Q4 (DT > 17.96%)	**0.72** [0.66, 0.77]	**0.72** [0.65, 0.75]	0.71 [0.65, 0.76]	33.3

The ranges in the first column indicate the percentage of dense tissue for each quartile group. Values are average AUCs corresponding to the three models. Data in brackets are 95% confidence intervals obtained with bootstrapping over 1000 iterations. The last column represents the percentage of interval cancers out of the total number of cases in each quartile. The DeLong test indicated no evidence of differences between any of the quartiles within each model (all *p* > 0.05)

*ERT* Extremely Randomized Trees, *CNN* Convolutional Neural Network, *pd* personal data, *im* image-extracted features

Bold values indicate the maximum AUC within each dense tissue quartile

### Subgroups according to cancer detection method

**Fig. 4:**
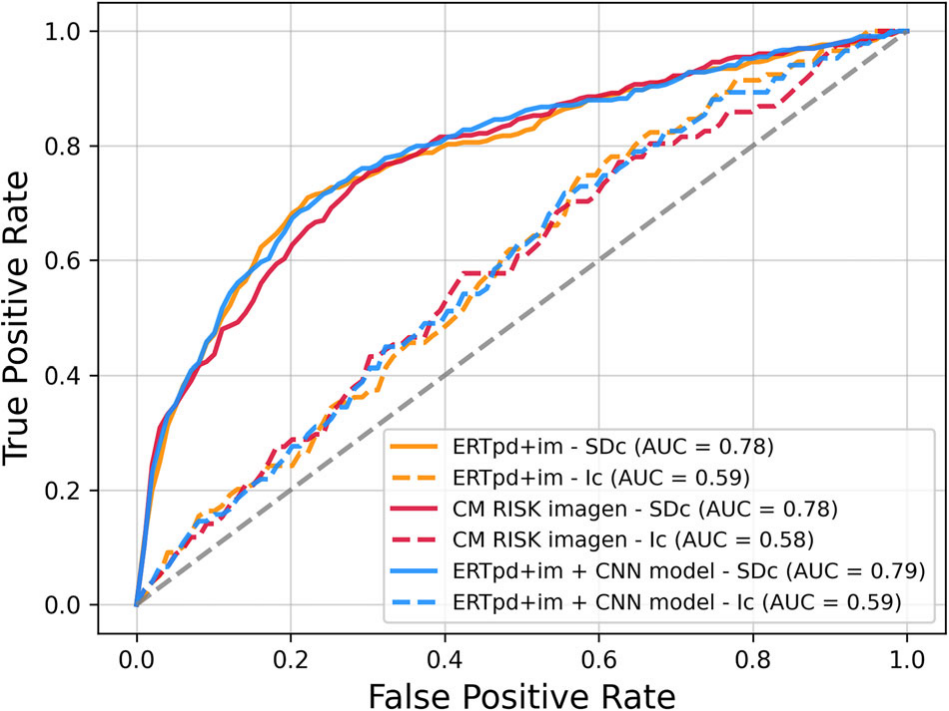
Receiver operating characteristic (ROC) curve of the ERTpd + im, CNN, and hybrid (ERTpd + im + CNN) models’ performance on the test set (pd = personal data, im = features extracted from images, ERT = Extremely Randomized Trees, CNN = Convolutional Neural Networks). Each model’s effectiveness is depicted by the mean AUC value, derived from the five test sets generated during the 5-fold cross-validation process. The curves represent the performance of the three models, distinguishing between screen-detected cancer (SDc) and interval cancer (Ic)

The performance evaluation of the models based on the method of cancer detection is illustrated in Fig. 4. The study sample included 98 interval cancers (23%) and 320 screen-detected cancers (77%). The risk estimation for the subgroup of screen-detected cancer cases resulted in significantly higher AUCs compared to interval cancer cases, with AUCs of 0.78 vs. 0.59 for ERT*pd + im*, 0.78 vs. 0.58 for CNN, and 0.79 vs. 0.59 for the hybrid model, ERT*pd + im* + CNN (all *p* < 0.001). No significant differences were observed among the three models within each subgroup.

### Feature importance

**Fig. 5:**
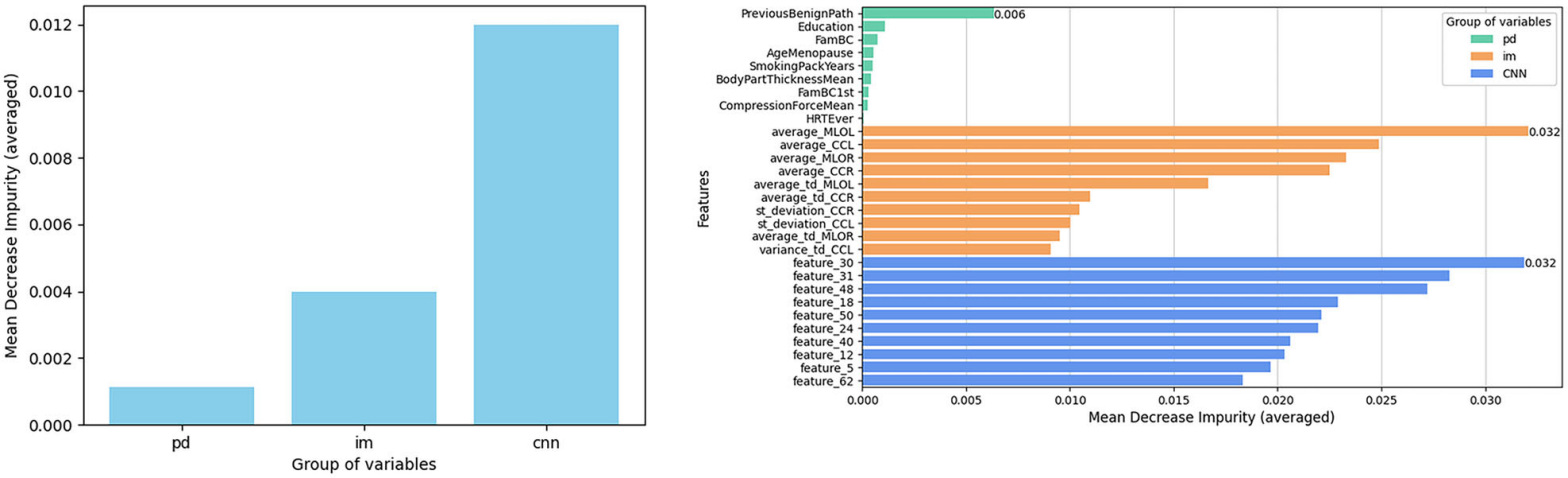
Feature importance analysis of the hybrid model. The mean decrease in impurity (MDI) is used to calculate the feature importance scores. Higher values indicate greater importance of the respective features in the model’s predictions. The data groups consist of: pd (personal data), im (features extracted from images), and CNN (features extracted from the neural network). **Left panel** Shows the averaged MDI for each group of variables. **Right panel** The top 10 most relevant features (or the maximum available) are shown for each data group

Feature importance analysis (Fig. 5) showed that the CNN model variables were, on average, the most influential in the hybrid model’s prediction, with an average MDI of 0.012 and a total MDI of 0.77. The mentioned features are not explicitly named because they are extracted from the penultimate layer of a neural network, making them inherently abstract.

Following this, image-extracted variables (*im*), had an average MDI of 0.004 and a total MDI of 0.22, contributing notably to the model. Since both the CNN features and the *im* variables are derived from mammography, the CNN may capture information within its features that overlaps with certain *im* variables. Lastly, personal data variables (*pd*) had the lowest importance, with an average MDI below 0.002, and a total MDI of 0.01. Within this group, the variable *Previous Benign Breast Disease* stood out with a notably higher value than the other variables in its group, followed by *Education*, No. of *first-degree relatives*, and *Age at menopause*.

**Fig. 6:**
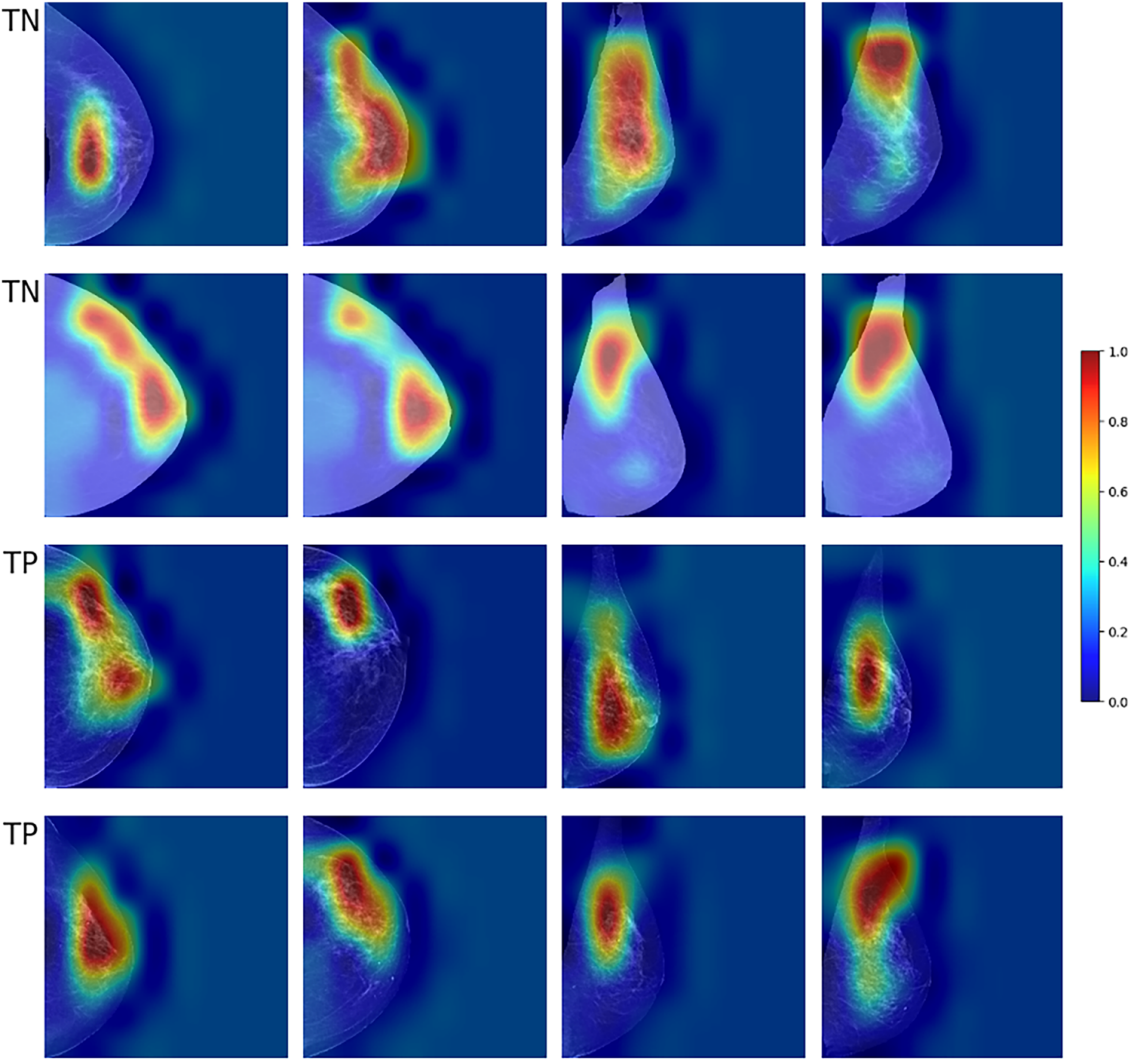
Grad-CAM visualizations of cases and controls correctly classified by the CNN model. The heatmaps show the mammographic regions the model focused on during risk prediction. Rows correspond to true negatives (TN, top two rows) and true positives (TP, bottom two rows). Color intensity reflects the level of model activation, with red indicating areas of strongest focus

To complement these findings and support CNN interpretability, Fig. 6 presents Grad-CAM visualizations from cancer cases and controls correctly classified by the CNN model. The heatmaps highlight image regions that contributed most to the model’s risk predictions. As the task involves future cancer risk prediction, these regions do not correspond to visible lesions, but rather to areas of DT or parenchymal patterns that the model relied on to generate its prediction.

In conclusion, the most substantial contribution to the model’s predictive power comes from the CNN group features, followed by the conventional image-derived features, and finally, the personal data features, showing the least importance.

## Discussion

In this study, we compared three machine learning models to predict the risk of developing breast cancer within two years. Our results showed that the combined model, which incorporated both clinical data and image features, achieved the highest average AUC of 0.75, indicating that both image and personal data provide valuable information, with a predominant focus on image data, as illustrated in the feature importance plot (Fig. 5). We also included Grad-CAM visualizations to illustrate which image regions the CNN model focused on during prediction, often highlighting DT and parenchymal patterns. The sub-models using clinical data and image data separately exhibited AUCs of 0.59 and 0.73, respectively, demonstrating the lower predictive capability of the clinical data model compared to the image features model (*p* < 0.05). While there was a slight trend suggesting lower model performance in higher breast density quartiles (Q3 and Q4), we did not find statistically significant differences among the density groups for any model (all *p* > 0.05). Our analysis also revealed that the distribution of interval cancers aligns with clinical expectations, with a higher occurrence in denser breasts due to the masking effect and interpretation difficulties in mammographic images.

In line with this, the models showed better performance for screen-detected cancer cases (*n* = 320) compared to interval cancer cases (*n* = 98), with AUCs of 0.79 vs. 0.59 for the combined model (*p* < 0.001). These findings suggest that all models were more effective in predicting screen-detected cancers. Contrary to what is often reported in the literature, where interval cancers are detected with greater sensitivity [34, 35], our models exhibited the opposite trend. This could be attributed to the low representation of interval cancer cases in our cohort, limiting the models’ ability to learn effectively from this subgroup, particularly since interval cancers are more common in denser breasts, which are more challenging to analyze.

Recent studies showed similar performance (Table 4), demonstrating the promise of artificial intelligence (AI) in breast cancer risk assessment. Comparing studies is challenging due to the different datasets, centers and devices. Despite this limitation, all studies share the use of mammograms and AI techniques, with follow-ups between 1 and 5 years. Some studies also include clinical data, although the latter varies depending on the research. Finally, we want to highlight that our proposed models demonstrated remarkable efficiency, achieving results comparable to other published studies, despite the challenge of having less data than existing reference models.

**Table 4:** Model performance of different breast cancer risk assessment studies

Study	Year	AUC (Personal data)^a^	AUC (Image)^a^	AUC (Hybrid)^a^	No. of women	Follow-up time (years)
Yala et al [19]	2021		0.78	0.80	70,811	2
Dembrower et al [39]	2020			0.65	14,034	3.6 ± 2
Zhu et al [35]	2021	0.62	0.66	0.66	6369	3
Arefan et al [40]	2019		0.73		226^b^	1.5 ± 0.7
Our proposed models	2024	0.59	0.74	0.75	2193	2

*AUC* area under the receiver operating characteristic curve

^a^ Indicates the type of data used in the model. A hybrid combines both personal and image data

^b^ The model was pre-trained on over a million images

Our study confirms that images and clinical data contain information with predictive capabilities, suggesting that AI models use mammograms as the main source for prediction, but also obtain complementary information from risk factors and other additional characteristics. This contribution could justify the superior predictive power of AI compared to traditional statistical procedures when assessing breast cancer risk, since they do not consider the images themselves. Research has demonstrated that AI techniques can learn complex relationships between different features of the images, which could explain a higher performance. In brief, statistical models are a valuable tool, although they could be combined with innovative technologies to increase performance even further.

The importance of the risk factors identified in our models aligns with findings from previous studies. In the combined model, the most predictive variables, aside from those related to imaging, were the presence of previous benign lesions [36], education level [37], and family history of breast cancer [38]. These risk factors have been widely studied and confirmed. Their importance in our models not only highlights their predictive power but also indicates the consistency of our results with established evidence.

The strengths of this study lie in the models’ robustness across varying breast density characteristics, consistency with prior research, and promising performance, particularly in screen-detected breast cancer cases. However, our study also has some limitations. First, the data comes from a single-center and two manufacturers, limiting its generalizability to other populations. Second, the relatively small sample size may affect the precision of the results. Third, the models exhibited lower performance for interval cancer cases. Finally, the data imbalance, with fewer cases than controls, can reduce model precision and increase the risk of bias. In the future, we expect to obtain more data to address the limitations of our current study and develop a specific model for interval cancer cases.

In conclusion, our study confirms that integrating both image and clinical data provides a significant advantage in predicting breast cancer risk compared to using either data type alone. The combined model demonstrated slightly superior predictive performance, especially for screen-detected cancers, while highlighting the challenges of interval cancer prediction. Despite limitations such as a single-center dataset and a relatively small sample size, our findings are consistent with prior research, highlighting the potential of combining AI with traditional risk factors to enhance breast cancer risk prediction. Future research should focus on acquiring larger, more diverse datasets and refining models to improve interval cancer detection, ultimately advancing the field of breast cancer risk assessment.

## Supplementary information

ELECTRONIC SUPPLEMENTARY MATERIAL

## Abbreviations

AUC: Area under the receiver operating characteristic curve
CNN: Convolutional Neural Networks
DT: Dense tissue
ERT: Extremely Randomized Trees
FFDM: Full-field digital mammography
MDI: Mean decrease impurity
