# Supplementary material for: Breast cancer risk assessment for screening: a hybrid artificial intelligence approach
Source: Eur Radiol. 2025 Sep 11;36(3):1932–42. doi: 10.1007/s00330-025-11980-9 (PMC12963263; doi:10.1007/s00330-025-11980-9)
Supplement: Supplementary file 1 — ELECTRONIC SUPPLEMENTARY MATERIAL [file 330_2025_11980_MOESM1_ESM.pdf]

Breast Cancer Risk Assessment for Screening: A Hybrid Artificial Intelligence Approach

ELECTRONIC SUPPLEMENTARY MATERIAL

Risk assessment models

ERT*pd+im* model

The Extremely Randomized Trees (ERT) model was implemented using the scikit-learn library (version 0.24.2; scikit-learn.org). The input data for this model included personal data (pd) and image-extracted features (im) (refer to Table 1 in the main document and Supplementary Table 2 in this document, respectively). Preprocessing involved filling in missing values and handling atypical entries, as detailed in the main document. The model parameters were set as follows: class_weight='balanced', criterion='entropy', max_depth=3, n_estimators=500, min_samples_split=10, and min_samples_leaf=10. Model evaluation was performed using a stratified 5-fold test.

CNN model

The Convolutional Neural Network (CNN) model was developed using TensorFlow (version 2.6.2; tensorflow.org) for the model and Albumentations (version 1.3.1; albumentations.ai) for data augmentation. The input data included mammography images, with each examination consisting of four projections (mediolateral oblique and craniocaudal views) per woman. Data preprocessing and augmentation were conducted as described in the Materials and Methods section. Data augmentation included transformations such as random brightness and contrast adjustments, Gaussian noise, blurring, random sized cropping, optical distortion, and grid distortion, with each original image being augmented twice.

The model architecture combined a pre-trained DenseNet121 as a feature extractor with a custom classifier. The classifier consisted of dense layers, batch normalization, dropout, Gaussian noise, and a final sigmoid activation. DenseNet121 extracted features from input images, which were then processed by the classifier to output predictions. Model evaluation was conducted using a 5-fold test.

ERT*pd+im* + CNN model

This hybrid model utilized both the scikit-learn (version 0.24.2; scikit-learn.org) and TensorFlow (version 2.6.2; tensorflow.org) libraries. Input data comprised personal data and image-extracted features (refer to Table 1 in the main document and Supplementary Table 2 in this document, respectively), along with the output from the penultimate layer of the CNN model, which provided a 64-dimensional feature vector. The model parameters included class_weight='balanced', criterion='entropy', max_depth=3, n_estimators=1000, min_samples_split=10, and min_samples_leaf=10. Evaluation of the model was performed using a 5-fold test.

Patient characteristics and data collection

Supplementary Table 1: Description of mammography acquisition parameters

Parameter	Controls (n = 1775)	Cases (n = 418)	Total
kVp	29.9 ± 2.05	30.62 ± 2.46	30.04 ± 2.15
Exposure time (msec)	821 ± 211	840 ± 258	825 ± 221
X-Ray tube current (mA)	86 ± 17	75 ± 19	83 ± 18
Exposure (mAs)	59 ± 19	59 ± 24	59 ± 20
Body part thickness (mm)	54.57 ± 11.16	53.88 ± 11.62	54.44 ± 11.25
Compression force (N)	73.87 ± 19.78	78.34 ± 21.6	74.72 ± 20.21
Organ dose (dGy)	54.57 ± 11.16	53.88 ± 11.62	54.44 ± 11.25

Note.- Data are mean value ± standard deviations.

Supplementary Table 2: List of variables extracted from images (*im*).

Geometric measurements
Area (No. of pixels) (DT)
Bounding box – coordinate x1 (DT)
Bounding box – coordinate y1 (DT)
Bounding box – coordinate y2 (DT)
Breast center – y-axis *
Centroid – x-axis (DT)
Perimeter (No. of pixels)
Statistical Properties of Pixels
Average pixel value *
Average pixel value * (DT)
Standard deviation of pixels *
Variance of pixels * (DT)
Invariant moments
Hu moments – No. 1
Hu moments – No. 4
Image/Acquisition Physical Properties
Compression force (N) †
Dense tissue (%) **
Body part thickness (mm) †

Note.- Unless otherwise indicated, variable refers to the entire breast. (DT) means the information corresponds to the dense tissue mask only. If not specified, the variable has been obtained with the *measure* package from *scikit-image* (version 0.17.2; scikit-image.org).
* The variable has been obtained with the *ndimage* package from the *SciPy* library (version 1.5.4; scipy.org).
** The variable has been obtained with CM-YNet, a deep learning model for dense tissue segmentation[23].
† The variable has been extracted from the *DICOMs*.

Supplementary Table 3: Characteristics of the variables extracted from images. Craniocaudal left view.

Variable	***Cases (n = 418)***	***Controls (n = 1,775)***
Area (No. of pixels) (DT)	3,136 ± 1,699	2,967 ± 1,649
Average pixel value	0.58 ± 0.20	0.57 ± 0.13
Average pixel value (DT)	0.73 ± 0.13	0.73 ± 0.09
Bounding box – coordinate x1 (DT)	3.38 ± 7.39	3.19 ± 7.62
Bounding box – coordinate y1 (DT)	24.46 ± 18.97	29.03 ± 21.10
Bounding box – coordinate y2 (DT)	240.68 ± 13.26	240.20 ± 13.07
Breast center – y-axis	136.82 ± 6.55	136.79 ± 6.18
Centroid – x-axis (DT)	72.20 ± 24.12	71.81 ± 24.52
Compression force (N)	78.34 ± 21.60	73.87 ± 19.78
Dense tissue (%)	13.98 ± 7.59	13.46 ± 7.62
Hu moments – No. 1	0.76 ± 0.87	0.92 ± 1.31
Hu moments – No. 4	5.14 ± 50.26	6.56 ± 85.53
Perimeter (No. of pixels)	1,385 ± 658	1,274 ± 612
Standard deviation of pixels	0.08 ± 0.04	0.09 ± 0.03
Body part thickness (mm)	53.88 ± 11.62	54.57 ± 11.15
Variance of pixels (DT)	0.005 ± 0.005	0.004 ± 0.003

Note.- Data are mean AUC ± standard deviations. Unless otherwise indicated, variable refers to the entire breast. (DT) means the information corresponds to the dense tissue mask only.

Supplementary Table 4: Characteristics of the variables extracted from images. Craniocaudal right view.

Variable	***Cases (n = 418)***	***Controls (n = 1,775)***
Area (No. of pixels) (DT)	3,305 ± 1,696	3,063 ± 1,627
Average pixel value	0.58 ± 0.21	0.57 ± 0.13
Average pixel value (DT)	0.73 ± 0.13	0.73 ± 0.09
Bounding box – coordinate x1 (DT)	3.41 ± 7.95	3.10 ± 7.57
Bounding box – coordinate y1 (DT)	21.25 ± 16.87	21.65 ± 17.41
Bounding box – coordinate y2 (DT)	240.14 ± 13.39	236.41 ± 16.40
Breast center – y-axis	134.94 ± 6.73	133.97 ± 6.12
Centroid – x-axis (DT)	71.56 ± 25.41	71.03 ± 25.09
Compression force (N)	77.98 ± 21.17	74.08 ± 20.07
Dense tissue (%)	14.68 ± 7.62	13.93 ± 7.68
Hu moments – No. 1	0.82 ± 1.05	0.87 ± 1.13
Hu moments – No. 4	4.81 ± 42.94	5.08 ± 66.11
Perimeter (No. of pixels)	1,438 ± 637	1,292 ± 593
Standard deviation of pixels	0.09 ± 0.04	0.09 ± 0.03
Body part thickness (mm)	53.47 ± 11.51	54.25 ± 11.07
Variance of pixels (DT)	0.004 ± 0.005	0.004 ± 0.003

Note.- Data are mean AUC ± standard deviations. Unless otherwise indicated, variable refers to the entire breast. (DT) means the information corresponds to the dense tissue mask only.

Supplementary Table 5: Characteristics of the variables extracted from images. Mediolateral oblique left view.

Variable	***Cases (n = 418)***	***Controls (n = 1,775)***
Area (No. of pixels) (DT)	2,523 ± 1,411	2,445 ± 1,357
Average pixel value	0.56 ± 0.23	0.55 ± 0.14
Average pixel value (DT)	0.73 ± 0.15	0.73 ± 0.09
Bounding box – coordinate x1 (DT)	5.94 ± 9.04	5.71 ± 8.82
Bounding box – coordinate y1 (DT)	55.83 ± 38.58	57.97 ± 39.92
Bounding box – coordinate y2 (DT)	244.13 ± 10.41	240.83 ± 12.16
Breast center – y-axis	150.16 ± 7.32	149.12 ± 7.09
Centroid – x-axis (DT)	72.04 ± 21.43	70.62 ± 21.51
Compression force (N)	83.58 ± 23.06	79.60 ± 21.52
Dense tissue (%)	12.44 ± 7.41	12.33 ± 7.28
Hu moments – No. 1	0.83 ± 1.03	0.85 ± 1.26
Hu moments – No. 4	3.98 ± 27.23	16.10 ± 205.04
Perimeter (No. of pixels)	1,142 ± 538	1,076 ± 506
Standard deviation of pixels	0.09 ± 0.04	0.10 ± 0.03
Body part thickness (mm)	57.90 ± 13.49	58.06 ± 12.69
Variance of pixels (DT)	0.005 ± 0.005	0.005 ± 0.003

Note.- Data are mean AUC ± standard deviations. Unless otherwise indicated, variable refers to the entire breast. (DT) means the information corresponds to the dense tissue mask only.

Supplementary Table 6: Characteristics of the variables extracted from images. Mediolateral oblique right view.

Variable	***Cases (n = 418)***	***Controls (n = 1,775)***
Area (No. of pixels) (DT)	2,544 ± 1,430	2,401 ± 1,367
Average pixel value	0.57 ± 0.22	0.56 ± 0.14
Average pixel value (DT)	0.73 ± 0.14	0.73 ± 0.10
Bounding box – coordinate x1 (DT)	6.98 ± 10.02	6.41 ± 10.05
Bounding box – coordinate y1 (DT)	57.53 ± 33.69	61.81 ± 32.38
Bounding box – coordinate y2 (DT)	242.60 ± 11.95	240.62 ± 12.91
Breast center – y-axis	149.78 ± 7.55	148.06 ± 7.17
Centroid – x-axis (DT)	72.49 ± 22.50	71.19 ± 21.71
Compression force (N)	83.86 ± 23.99	79.48 ± 23.23
Dense tissue (%)	12.69 ± 7.48	12.30 ± 7.46
Hu moments – No. 1	0.76 ± 0.87	0.87 ± 1.31
Hu moments – No. 4	1.61 ± 10.91	7.23 ± 70.72
Perimeter (No. of pixels)	1,126 ± 511	1,036 ± 490
Standard deviation of pixels	0.09 ± 0.04	0.10 ± 0.03
Body part thickness (mm)	57.36 ± 13.49	57.11 ± 12.44
Variance of pixels (DT)	0.005 ± 0.005	0.004 ± 0.003

Note.- Data are mean AUC ± standard deviations. Unless otherwise indicated, variable refers to the entire breast. (DT) means the information corresponds to the dense tissue mask only.
